# One Health: governance and regulatory framework for antimicrobial use in Malawi

**DOI:** 10.1016/j.soh.2025.100119

**Published:** 2025-07-19

**Authors:** Amos Lucky Mhone, Dishon M. Muloi, Arshnee Moodley

**Affiliations:** a Health Program, International Livestock Research Institute, Nairobi, Kenya; b Department of Veterinary and Animal Sciences, Faculty of Health and Medical Science, University of Copenhagen, Frederiksberg C, Denmark; c Institute of Infection, Veterinary and Ecological Sciences, University of Liverpool, Liverpool, United Kingdom

**Keywords:** Antimicrobial use, Regulatory framework, Gaps, Governance, Policy

## Abstract

**Background:**

Antimicrobial resistance (AMR) poses a global threat to both human and animal health, associated with widespread use of antimicrobials across sectors. In low- and middle-income countries (LMICs) such as Malawi, weak regulatory frameworks and limited enforcement capacity contribute to inappropriate use of antibiotics. This study examined the governance and regulatory frameworks for antimicrobial use (AMU) in Malawi's agricultural sector, identified regulatory gaps, and offers recommendations to antimicrobial stewardship.

**Methods:**

A qualitative approach was used, combining a review of policy and legal documents with semi-structured stakeholder interviews. Relevant policies and laws were sourced from government databases, the Food and Agricultural Organisation of the United Nations' (FAO) FAOLEX and AMR-LEX databases, and other publicly available resources. The FAO's legal assessment methodology was used to evaluate the policy landscape across nine key thematic areas: (1) veterinary medicinal products, (2) animal health and production practices to prevent animal diseases in terrestrial and aquatic animals, (3) feed registration, (4) pesticides, (5) food safety, (6) environment, soil and waste, (7) water quality, (8) plant health, and (9) institutional coordination. Stakeholder interviews with representatives from relevant government ministries and regulatory bodies validated findings from the document review and provided additional insight into governance challenges. A One Health governance mapping exercise was conducted to identify key institutional actors, assess their role in AMR/AMU governance, and evaluate inter-institutional relationships using social network analysis.

**Results:**

The analysis identified 522 policies relevant to AMU in agriculture, with most addressing aquatic animal health (11.3 %, *n* = 59), food safety (10.9 %, *n* = 57) and animal feed (10.9 %, *n* = 57). Several critical regulatory gaps were identified, including the absence of a legal definition for “antimicrobials,” a national essential veterinary medicines list, and standardized veterinary treatment guidelines. Additionally, there are no restrictions on the use of critically important antimicrobials for human health in veterinary settings, minimal oversight of antimicrobial-medicated feed, and no established protocols for on-farm antimicrobial disposal. Stakeholder mapping revealed limited knowledge sharing among institutions and a dependence on international donors for AMR/AMU-related activities, raising concerns about the sustainability of current initiatives. Malawi also lacks an integrated AMR and AMU monitoring system, a national prioritised AMR research agenda, and clear targets for reducing AMU in animals.

**Conclusion:**

To address these gaps, we recommend that Malawi: (1) establish a comprehensive AMR and AMU monitoring program, (2) update existing regulations to provide clear definitions and classification of veterinary antimicrobials, (3) develop and implement national veterinary treatment guidelines, (4) restrict non-therapeutic AMU, (5) enhance regulatory oversight of medicated feed, (6) strengthen One Health coordination mechanisms, (7) promote stakeholder collaboration, and (8) secure sustainable, nationally driven funding. Implemention of these measures will enhance antimicrobial stewardship, reduce AMU, mitigate the spread of AMR, and support the long-term sustainability of agricultural production in Malawi.

## 1 Introduction

Antimicrobial resistance (AMR) is a global health challenge that threatens the effectiveness of treatments for infectious diseases in both humans and animals [1]. The increasing use of antimicrobials in agriculture significantly contributes to the development of resistant microorganisms, which can spread through food chains, the environment, and direct human–animal interactions [2]. In low- and middle-income countries (LMICs), where regulatory frameworks are lacking or poorly enforced, addressing antimicrobial use (AMU) in agriculture is critical to mitigating AMR and safeguarding public health [[3], [4], [5]].

Malawi's growing demand for livestock products has led to increased use of veterinary antimicrobials to promote productivity through treatment and prevention of livestock diseases [6]. The absence of a comprehensive regulatory framework and governance mechanisms regulating AMU in agriculture poses risks of antimicrobial misuse, which can accelerate the emergence and spread of resistance [7,8]. Understanding the current regulatory landscape and identifying gaps is essential to developing targeted interventions that promote responsible antimicrobial stewardship while supporting the agricultural sector's productivity and sustainability.

Globally, the One Health approach, which recognizes the interconnectedness of human, animal, and ecosystem health, has become important for AMR mitigation efforts [9,10]. Effective governance of AMU requires coordination across diverse stakeholders, including government agencies, veterinary professionals, farmers, and the pharmaceutical industry [11]. International organizations such as the World Health Organization (WHO), the Food and Agriculture Organization of the United Nations (FAO), and the World Organisation for Animal Health (WOAH) have established guidelines to support countries in developing integrated AMR action plans. The FAO's methodology for assessing AMR-relevant legislation in the food and agriculture sector provides a comprehensive framework for evaluating the effectiveness of existing regulations and identifying areas for improvement [12].

Despite global efforts to address AMR, many LMICs, such as Malawi, continue to face governance challenges. Fragmented regulatory frameworks, limited enforcement capacity, and inadequate stakeholder coordination often hinder the effective implementation of AMU policies [3,4,13]. Additionally, over-reliance on international donors for funding AMR initiatives can create financial instability, making it difficult to sustain long-term interventions [9]. This study aimed to analyze the governance and regulatory framework for AMU in Malawi's agricultural sector, identify existing gaps, and provide recommendations for strengthening AMU policies. By comparing Malawi's regulations with those of other LMICs that align with international recommended best practices, this study seeks to contribute to the development of cost-effective regulatory frameworks and governance structures that promote responsible AMU, help mitigate the spread of AMR, and promote the sustainability of Malawi's agricultural sector.

## 2 Methods

### 2.1 Study design

A desk-based qualitative study was conducted to identify, collect and synthesize existing policies, laws and regulations relevant to AMU in the agricultural sector in Malawi, along with institutional arrangements linked to AMU governance. This study was carried out between May 2023 and June 2023. Ethical approval was obtained from the International Livestock Research Institute-Institutional Research Ethics Committee (ILRI-IREC) (project reference: ILRI-IREC2022-33), and research permit was obtained from the Malawi Government's Department of Animal Health and Livestock Development (DAHLD) (project reference: DAHLD/AHC/01/2023/07).

### 2.2 Document search strategy

The review utilized comprehensive searches on databases such as Google Scholar and PubMed, focusing on grey literature, government gazettes, and official publications. Other sources included government websites such as the Malawi Ministry of Agriculture, Irrigation and Water Development (MoAIWD), as well as the FAO's FAOLEX and AMR-LEX databases. FAOLEX is one of the most comprehensive global repositories of national laws, regulations, and policies related to food, agriculture, and natural resources. Maintained by the FAO's Development Law Service, it offers free access to abstracts, indexing information, and in many cases, full-text legal documents from around the world [14]. Building on this foundation, AMR-LEX was launched by FAO in September 2022. AMR-LEX is a publicly accessible legal database that specifically focuses on legal and policy instruments related to AMR and AMU in the food and agriculture sectors. It adopts a One Health approach and includes both AMR-specific legislation and broader sectoral laws that influence AMU. The platform also provides country and regional profiles, enabling users to explore regulatory landscapes and compare approaches across contexts [15].

The search was guided by keywords such as “AMU policies,” “legislation,” “laws,” “strategy,” “governance,” and “regulation”. Documents were included if they provided information on AMU policies, legislations, regulations, strategies, or described the roles of regulatory bodies. In this paper, “policy” is used generally to refer to legislation, laws, regulations, strategies, codes of practice, guidelines, and quality assurance programs. We adopted the definitions by FAO for the policy documents included in the study, as summarised in Table 1. Data extraction focused on key aspects such as content, regulatory bodies involved, publication years, establishment dates, and subsequent amendments.

**Table 1:** Definition of policy documents included in the study.

Policy document	Definition
Policy	The stated objectives that a government seeks to achieve, reflected in decisions made by individuals, organizations, or governments directed at addressing specific issues
Strategy	A detailed plan outlining how a policy will be implemented to achieve its goals, for example, national action plans on antimicrobial resistance
Legislation	Official documents enacted by a national legislative or parliamentary body; this includes laws, authorizations or other official formal directives
Law	Legally binding obligations established by a competent authority; the term “law” is used when referring to specific enacted legal instruments
Regulation	Legally binding rules issued by a government agency or ministry, often providing specific detail on how to implement broader legislation
Regulatory framework	A national system of legislation encompassing laws, legislation, policies, strategies, and the institutions responsible for their implementation

### 2.3 Document analysis

The FAO methodology for analysing AMR-relevant legislation in the food and agriculture sector was used to evaluate policy documents. This methodology constitutes nine key areas of assessment: (1) veterinary medicinal products, (2) animal health and production practices to prevent animal diseases in terrestrial and aquatic animals, (3) feed registration, (4) pesticides, (5) food safety, (6) environment, soil and waste, (7) water quality, (9) plant health, and (9) institutional coordination [12]. A comprehensive analysis was conducted to identify gaps and areas for improvement in Malawi's AMU regulatory framework. This data was organized into tables and visualized using *Microsoft Excel* and *R* software (version 4.4.2).

### 2.4 Stakeholder interviews

Semi-structured interviews were conducted with stakeholders from Malawi to validate findings from the literature review. Participants included representatives from relevant ministries and regulatory bodies, namely, the Pharmacy, Medicines, Regulatory Authority (PMRA) and the DAHLD. The interviews focused on the state and effectiveness of the current regulatory framework, identifying gaps in enforcement and highlighting areas for improvement.

### 2.5 One Health governance mapping

A list of state agencies and private organizations working on AMU in Malawi was compiled with the help of Malawi Antimicrobial Resistance National Coordinating Centre (AMRNCC). A semi-structured questionnaire was administered to representatives from respective organizations (Supplementary material 1). The interviews focused on their involvement in AMR/AMU work, partners they work with, funders, and challenges to their partnerships. For the analysis, stakeholder organizations were classified based on sector (governmental or non-governmental), primary One Health focus area (animal, human, or environmental health), organizational type (research, policy, or administration), and their main AMR-related activities (education and awareness, surveillance and research, infection prevention and control, optimal use of antimicrobials, and investment and sustainability). Stakeholder relationships were transcribed into a single network matrix for network analysis using *Kumu* (version 9.3.2) and displayed using Social Network Visualizer (version 3.2). Degree centrality, betweenness centrality, closeness centrality and reach efficiency were used to assess the influence of each organization on the entire network. All centrality scores were normalized (scaled between 0 and 1) to allow comparison across stakeholders of varying network sizes and densities (Table 2) [9].

**Table 2:** A brief description of network analysis metrics assessed for AMR/AMU governance in Malawi.

Metric	Description	Formulas
Degree centrality	This metric counts the number of connections an element has, i.e., elements with high degree are the local connectors/hubs, but aren't necessarily the best connected to the wider network	$C_{D}\left(v\right)=\deg\left(v\right)$ Where $C_{D}\left(v\right)$ is the degree centrality of node ⁡(v) and deg⁡(v) is the number of edges (links) incident to node ⁡(v) [16]
Betweenness centrality	This metric measures how many times an element lies on the shortest path between two other elements, i.e., elements with high betweenness have more control over the flow of information and act as key bridges within the network; they can also be potential single points of failure	$C_{B}\left(v\right)=\sum_{s\neq v\neq t}\frac{\sigma_{st}\left(v\right)}{\sigma_{st}}$ Where $C_{B}\left(v\right)$ is the betweenness centrality of node v; $\sigma_{st}$ is the total number of shortest paths from node *s* to node *t*; and $\sigma_{st}\left(v\right)$ is the number of those paths that pass-through node *v*. This measure highlights nodes that serve as key bridges in the network [17]
Closeness centrality	This metric measures the distance each element is from all other elements, i.e., elements with high closeness can spread information to the rest of the network most easily and usually have high visibility into what is happening across the network	$C_{c}\left(v\right)=\frac{1}{\sum_{t\neq v}d\left(v,t\right)}$ where $C_{c}\left(v\right)$ is the closeness centrality of node *v*, and d(v,t) is the shortest distance between node *v* and node *t*. Nodes with higher closeness are structurally more accessible to others [18].
Reach efficiency	Reach efficiency normalizes reach by dividing it by size (number of neighbours), i.e., elements with high reach efficiency are less connected but gain more exposure through each direct relationship	$RE\left(v\right)=\frac{R\left(v\right)}{T\left(v\right)}$ where RE(v) is the reach efficiency of node *v*, R(v) is the total number of nodes reachable from *v*, and T(v) is the total number of steps or distances taken to reach those nodes [[18], [19]].

Abbreviations: AMR, antimicrobial resistance; AMU, antimicrobial use.

## 3 Results

### 3.1 Mapping antimicrobial use policy documents in the Malawian agricultural sector

**Fig. 1:**
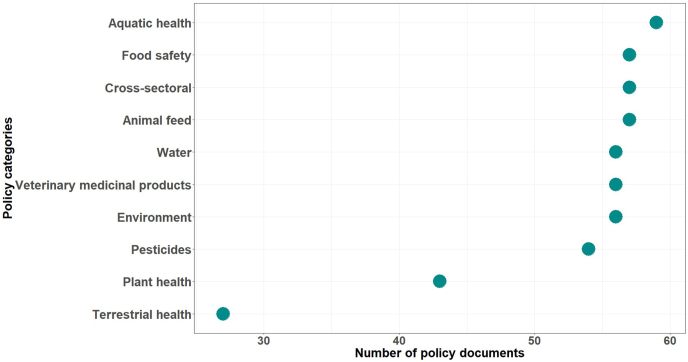
Categorization of laws related to antimicrobial use in the Malawian agricultural sector.

A total of 522 sector-specific and cross-sectoral policy documents were identified in the Malawi agricultural sector. Most of these were related to aquatic animal health (11.3 %, *n* = 59), food safety (10.9 %, *n* = 57), and animal feed (10.9 %, *n* = 57), while fewer were related to terrestrial animal health and production (5.2 %, *n* = 27), and plant health (8.2 %, *n* = 43) (Fig. 1).

### 3.2 Gaps in the antimicrobial use regulatory framework in the Malawian agricultural sector

#### 3.2.1 Governance and coordination

Malawi lacks a prioritized AMR research agenda, which is a strategic plan that outlines the national research goals and priorities. This absence limits coordination and strategic direction for AMR-related research. The country also lacks an integrated AMU monitoring program, including mechanisms for systematic evaluation, periodic revision and sustainable financing. Furthermore, no benchmarking system exists for antibiotic prescriptions by veterinarians (Supplementary material 2). The regulatory framework does not define coordination mechanisms among institutions responsible for food safety governance (Supplementary material 2).

#### 3.2.2 Antimicrobial regulation

The existing legislation defines medicines in general; however, it does not specifically define key terms such as “antimicrobials” or any other variations of this term. Malawi also lacks a national essential veterinary medicines list and veterinary treatment guidelines (Supplementary material 2).

#### 3.2.3 Antimicrobial use

There are no legal restrictions on the use of certain critically important antimicrobials for human medicine in animals, nor are there provisions mandating their use as a last resort in animals. Additionally, there is no regulation on off-label or extra-label use of veterinary products. Farmers are not legally required to return unused or expired antimicrobials, maintain AMU records, or report usage. There is also no requirement for veterinary supervision in the administration of antimicrobials. Training on prudent AMU, biosecurity, and regulatory updates is not mandated. Furthermore, there are no risk-based protocols for disease prevention, nor is antimicrobial susceptibility testing required before prescription (Supplementary material 2).

#### 3.2.4 Antimicrobial medicated feed

The regulatory framework does not specifically restrict the sale, import, and use of antimicrobial-medicated feed. There is no requirement for prescriptions or oversight of its use, nor are there legal obligations for record-keeping. Regulations to prevent contamination between medicated and non-medicated feed are absent, as is specific legislation addressing AMU in aquaculture (Supplementary material 2).

#### 3.2.5 Antimicrobial waste and residues

The current regulatory framework does not provide guidance on the use of manure from treated animals as fertilizer, nor does it restrict the use of milk or animal by-products from such animals for feed. Guidelines for wastewater reuse in agriculture and regulations concerning antibiotic residues in effluents and waste are also lacking (Supplementary material 2).

### 3.3 Mapping AMU governance in Malawi

**Fig. 2:**
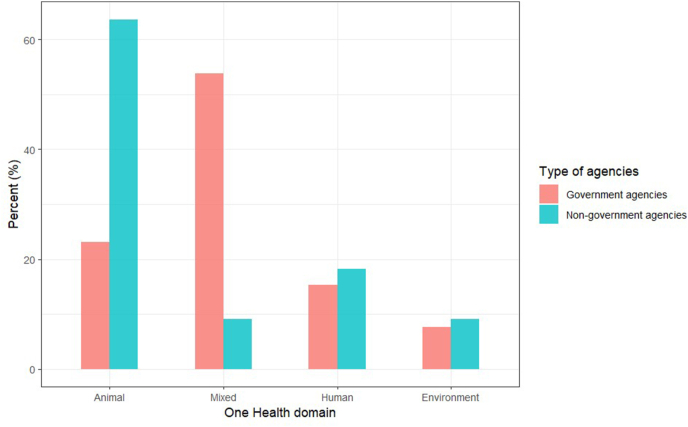
Summary of antimicrobial use governing agencies in Malawi based on their One Health scope.

A total of 24 organizations were identified, with 61 directed and mutual relationships. Most were governmental agencies (54.2 %, *n* = 13), followed by non-governmental organizations (NGOs) (45.8 %, *n* = 11). Regarding their One Health scope, the majority focused on the animal health sector (41.7 %, *n* = 10), followed by multi-sectoral (33.3 %, *n* = 8), human health (16.7 %, *n* = 4), and environment (8.3 %, *n* = 2) (Fig. 2).

### 3.4 Social network analysis

#### 3.4.1 Degree centrality

**Fig. 3:**
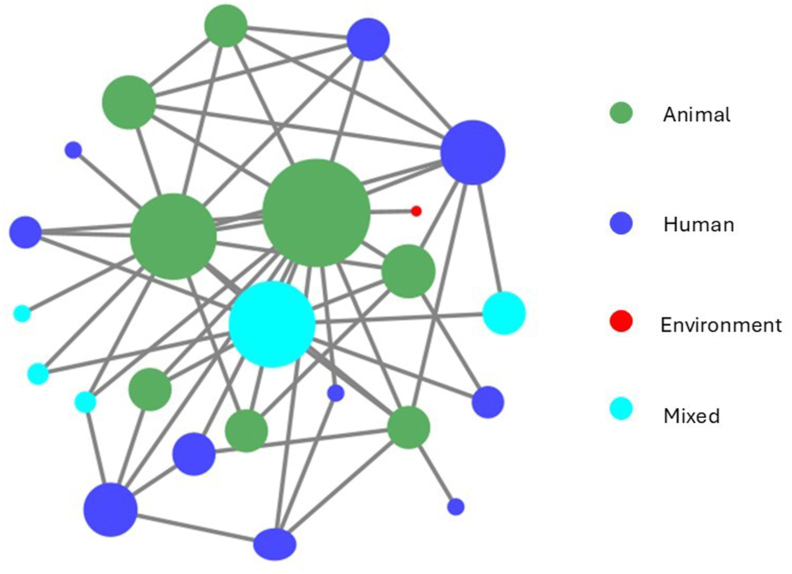
Social network analysis indicating antimicrobial use stakeholders in Malawi working in human, animal and environmental health sectors. Organization colour represents primary focus. Size is positively correlated to the weighted number of connections (weighted degree).

The Ministry of Agriculture (specifically DAHLD) held the highest degree of centrality with 15 connections, indicating its pivotal role in AMU governance. The AMRNCC and the Ministry of Health followed with 14 and 13 connections, respectively, reflecting their central roles in One Health governance (Fig. 3, Supplementary material 3).

#### 3.4.2 Betweenness centrality

The Ministry of Agriculture also ranked highest (0.448), acting as a key intermediary across sectors. The Ministry of Health (0.257) and AMRNCC (0.254) followed. Academic institutions played secondary roles, with lower centrality values (0.078 and 0.044), suggesting limited influence on decision-making. The national medicine regulatory authority (0.043) and international organizations (0.042) facilitated connections between government and non-state actors. Civil society and community-based organizations had zero betweenness centrality, reflecting weak integration in AMU governance networks (Supplementary material 3).

#### 3.4.3 Closeness centrality

AMRNCC scored highest (0.732), followed by the Ministry of Health (0.688) and Ministry of Agriculture (0.679), highlighting their strong positioning for information dissemination. FAO (0.591) and the Central Veterinary Laboratory (0.562) also emerged as well-connected technical actors. Academic institutions were moderately connected, while civil society organizations were least integrated (Supplementary material 3).

#### 3.4.4 Reach efficiency

Community-based organizations (0.292) and civil society organizations (0.271) exhibited the highest reach efficiency, suggesting they are well-positioned to disseminate information despite limited direct connections. Health NGOs also showed relatively high efficiency. In contrast, key government actors (Ministry of Health, Ministry of Agriculture, AMRNCC) had low efficiency scores, indicating centralization and limited ability to leverage broader partnerships (Supplementary material 3).

### 3.5 Key constraints in the network

Interviews highlighted three major constraints in Malawi's One Health governance system: (1) limited and fragile institutional relationships, (2) over-reliance on external funding, and (3) competing national priorities. Partnerships tend to be limited to familiar institutions, with most knowledge-sharing occurring during externally funded events such as World Antimicrobial Awareness Week (WAAW). Community-based organizations expressed a desire for stronger collaboration with government and NGOs. AMR-related initiatives are often deprioritized in national budgets—for example, agriculture investments such as Malawi's Farm Input Subsidy Programme (FISP) receive more consistent funding than animal health or AMU interventions.

## 4 Discussion

An integrated AMU monitoring program enables simultaneous tracking of antimicrobial consumption across human, animal and environmental health sectors, supporting the timely detection and analysis of AMU patterns [20,21]. While several LMIC countries, including Malawi, Ghana, India, Nigeria, and Nepal [22,23] have developed AMU monitoring initiatives, these efforts are often concentrated within the healthcare sector. Key challenges to establishing a fully integrated AMU monitoring system in LMICs include: (1) fragmented health information systems, which complicates comprehensive national-level analyses [3,4]; (2) lack of standardized data collection tools; (3) variability in data quality due to inconsistent reporting and collection practices; (4) limited funding for AMU surveillance activities; and (5) insufficient human resource capacity for data management and analysis [24,25]. Building on its existing health information infrastructure, Malawi could further strengthen AMU monitoring by: (1) developing user-friendly, locally adaptable AMU data collection tools; (2) leveraging and linking existing systems across sectors; (3) engaging stakeholders from government, healthcare, animal health, academia, and the pharmaceutical industry in the system design and governance; and (4) investing in professional training for AMU data collection, analysis, and interpretation.

The development of a prioritized AMR research agenda, coupled with defined national AMU reduction targets, offers an opportunity to further operationalize Malawi's National Action Plans (NAPs). At the 2024 United Nations General Assembly, global leaders committed to reducing inappropriate antibiotic use by 20 % in humans and significantly in animals [26]. Countries such as Belgium, Denmark, France, Malta, Slovenia, and the United Kingdom have incorporated similar targets into their national AMR action plans [27]. While recognizing resource and capacity constraints unique to LMICs, establishing tailored, measurable AMU reduction targets in Malawi could strengthen its NAP implementation. Complementary actions might include strengthening surveillance, engaging a broad range of stakeholders, and promoting interventions such as AMU education, clinical guideline development, and cross-sectoral coordination.

The absence of standardized and precise definitions for terms such as “antimicrobials” and “antimicrobial agents” presents major challenges across Malawi's regulatory framework. Legally, it creates ambiguity in the scope of legislation, leading to uncertainty over which substances fall under regulatory control. This can result in loopholes, inconsistent enforcement, and regulatory overlap. Technically, inconsistent definitions hamper data collection and surveillance efforts, as various actors may interpret and report AMU and AMR differently. This reduces the reliability, comparability and utility of national and international data. Practically, the ambiguity creates confusion among veterinarians, paraprofessionals, and drug sellers, increasing the risk of inappropriate or excessive AMU. It also enables the expansion of unregulated or informal markets [28]. Additionally, the lack of harmonized definitions across sectors hinders multi-sectoral coordination and weakens regional and global One Health responses to AMR. Updating Malawi's regulatory framework to include clear and harmonized definitions is therefore essential for improving governance, surveillance, and stewardship.

Malawi has demonstrated its commitment to AMR containment through the development of its NAPs and participation in regional and international initiatives. Further strengthening of the veterinary medicines regulatory framework could advance these efforts by ensuring the availability of safe, effective and quality-assured antimicrobials. Such frameworks also facilitate innovation by providing predictability for manufacturers and researchers [29,30]. In Malawi, the development of a national essential veterinary medicines list represents an opportunity to support harmonized prescribing. To strengthen the regulatory landscape, Malawi could consider (1) establishing a veterinary medicinal product classification system that reflects risk-based use, (2) developing and maintaining a national veterinary essential medicines list, and (3) prioritizing stewardship of critically important antimicrobials through targeted access and use policies. These actions would align Malawi's regulatory environment with international best practices while supporting innovation and animal health.

Although Malawi's regulatory framework includes provisions that aim to restrict access to antimicrobials, in practice, antibiotics remain widely accessible, including through informal markets and over-the-counter sales in veterinary drug shops [31,32]. This unregulated access undermines efforts to ensure responsible use and contributes to inappropriate or excessive AMU. However, this challenge coexists with a contrasting issue: in rural or marginalised areas, farmers struggle with limited or no access to veterinary professionals and/or veterinary medicines [32]. Restricting antimicrobial access through legislation is a necessary step toward better regulation. However, such measures must be carefully balanced with the need to ensure equitable access to animal health care. Without parallel investments in veterinary service delivery, stricter controls could unintentionally prevent farmers from obtaining timely treatment, risking animal health and livelihoods. To avoid this, AMU legislation must be complemented by expanded access to quality veterinary care. This includes training and deploying more veterinary paraprofessionals, establishing mobile and community-based veterinary services, and creating incentives to retain professionals in underserved areas. Encouragingly, Malawi has established its own veterinary school, and approximately five cohorts of 15 students each have graduated by 2024. This is a promising step toward strengthening the veterinary workforce [33,34].

Addressing non-therapeutic uses of medically important antimicrobials, such as for growth promotion and in medicated feed, represents another opportunity for Malawi to strengthen AMR mitigation efforts. While regulatory restrictions in this area are currently limited, learnings from Namibia, which introduced comprehensive bans and monitoring frameworks, could inform a phased, context-appropriate approach [35]. Possible steps may include (1) clarifying permissible AMU under national legislation, (2) strengthening compliance monitoring systems, (3) strengthening veterinary oversight mechanisms, and (4) supporting awareness-raising and education campaigns for farmers and feed producers.

Environmental dimensions of antimicrobials remain underdeveloped globally, including in Malawi. Problems include the absence of legal provisions for the disposal of unused antimicrobials, the use of manure from treated animals, or the consumption of milk and animal by-products from such animals. The absence of environmental AMU safeguards increases the risk of antimicrobial residues entering soil and water systems, potentially contributing to AMR transfer [36,37]. To mitigate this, Malawi could consider: (1) developing specific legal provisions on antimicrobial waste management, (2) regulating the reuse of manure, by-products and wastewater, (3) mandating training programs on prudent AMU, environmental protection, and compliance requirements for relevant stakeholders.

Sustained political will and strengthened coordination mechanisms are key to reducing AMU while ensuring productivity in the agriculture sector [26]. Limited institutional knowledge-sharing and fragmented relationships between government, NGOs, and community-based organizations constrain collective responses to AMR [38,39]. While initiatives such as WAAW help raise awareness, their episodic nature may limit long-term impact [40]. Moreover, reliance on international donor funding introduces uncertainty into the continuity of AMR programmes, particularly in the veterinary sector, which may receive less domestic prioritization compared to crop production programmes [9]. Like many LMICs, Malawi faces significant fiscal constraints and competing national priorities, including infectious disease control, food insecurity, and infrastructure development, making it difficult to justify sustained investments in AMR [41]. As a result, AMR activities remain reliant on external donor support, raising concerns about long-term continuity. To address this, political engagement and high-level advocacy are essential to elevate AMR on the national agenda and promote domestic ownership. Key enablers include developing a costed NAP on AMR, integration of AMR activities into health and agriculture sector budgets, and generation of local evidence on the cost-effectiveness of AMR interventions. Embedding AMR into national development plans and medium-term expenditure frameworks can elevate alignment with national priorities and reduce vulnerability to shifting donor priorities. Countries such as Ghana have successfully taken this approach [42]. While the transition from donor-driven to domestically financed AMR responses is complex, it is a critical step toward building resilient and sustainable health systems. Strengthening governance could involve: (1) establishing formalized partnerships and memoranda of understanding that clarify stakeholder roles and promote alignment, (2) prioritizing AMR activities in national budgets to reduce financial dependency on external donors, and (3) supporting ongoing collaborations through joint training, policy dialogues, and cross-sectoral working groups. These actions can create a more resilient and coordinated AMR response, rooted in national ownership and long-term sustainability.

## 5 Limitations

This study relied exclusively on publicly accessible online documents, which may not include internal or unpublished policies. As a result, some relevant legislation or institutional practices may have been excluded, potentially limiting the comprehensiveness of the analysis.

## 6 Conclusion

This study identifies key opportunities to strengthen Malawi's legislation and governance frameworks for AMU in the agricultural sector. These include the development of an integrated AMU monitoring system, the establishment of a national AMR research agenda, and clearly articulated regulations for veterinary antimicrobials. By building on its existing achievements and institutional frameworks, Malawi can further strengthen antimicrobial stewardship and reduce AMR risks. Prioritizing actions such as regulating the use of critically important antimicrobials, improving environmental safeguards, and enhancing stakeholder collaborations will be essential to advancing One Health goals. Strengthening domestic funding mechanisms and embedding AMR priorities into the national planning process can further promote sustainability and resilience across sectors.

## CRediT authorship contribution statement

**Amos Lucky Mhone:** Writing – review & editing, Writing – original draft, Visualization, Validation, Supervision, Software, Project administration, Methodology, Investigation, Formal analysis, Data curation, Conceptualization. **Dishon M. Muloi:** Writing – review & editing, Writing – original draft, Visualization, Validation, Supervision, Software, Project administration, Methodology, Investigation, Formal analysis, Data curation, Conceptualization. **Arshnee Moodley:** Writing – review & editing, Writing – original draft, Supervision, Resources, Project administration, Methodology, Funding acquisition, Conceptualization.

## Funding

This work was supported by the CGIAR One Health initiative “Protecting Human Health Through a One Health Approach” and the CGIAR Science Programme “Sustainable Animal and Aquatic Foods (SAAF)”, which are supported by contributors to the CGIAR Trust Fund (https://www.cgiar.org/funders/).

## Declaration of competing interest

The authors declare that they have no known competing financial interests or personal relationships that could have appeared to influence the work reported in this paper.
