# Supplementary material for: One Health: governance and regulatory framework for antimicrobial use in Malawi
Source: Sci One Health. 2025 Jul 19;4:100119. doi: 10.1016/j.soh.2025.100119 (PMC12362691; doi:10.1016/j.soh.2025.100119)
Supplement: Multimedia component 1 [file mmc1.docx]

**Supplementary Materials**

**Supplementary Material 1: Questionnaire for mapping AMR/AMU governance stakeholders in Malawi**

| 1. Questionnaire ID/Barcode |  |
| --- | --- |
| 1. Date of Survey | DD/MM/YYYY |
| 1. Enumerator’s name |  |
| 1. Name of the respondent (Optional) |  |
| 1. Name of the Institution |  |
| 1. Position of the respondent in the institution |  |
| 1. What activities is the stakeholder institution involved in, in relation to the AMR NAPs objectives and strategies? | 🞏1 Education and awareness  (Specify)  🞏2 Surveillance and Research  (Specify)  🞏3 Infection, prevention, and control  (Specify)  🞏4 Optimal Use  (Specify)  🞏5 Investment and sustainability  (Specify) |
| 1. To which technical working groups are your activities aligned with? | 🞏1 Education and awareness  🞏2 Surveillance and Research  🞏3 Infection, prevention, and control  🞏4 Optimal Use  🞏5 Investment and sustainability |
| 1. Who funds the budget for the activities? | 🞏 (Specify) |
| 1. Can you name the stakeholder you interact with? Or the activities mentioned in 6 | 🞏 (Specify) |
| 1. What are the strengths of the partnerships/ interactions with the stakeholders mentioned in 10?? |  |
| 1. What are the challenges the stakeholder faces in sharing AMR/AMU information? | 🞏 (Specify) |

**Supplementary Material 2: Gap Analysis of Antimicrobial Use-Relevant Legislation in the Food and Agriculture Sector in Malawi**

|  | **Legal Area** | **Policy/Law/Regulation Evidence** | **Documentation** |
| --- | --- | --- | --- |
|  | **Analysis of national legislation on veterinary medicinal products (antimicrobials)** |  |  |
|  | **Definition** |  |  |
|  | Is there a definition for VMP? Are VMPs part of a broader definition of medicine products?  *Part of a broader definition* | “Medicinal product” means any substance or combination of substances which may be administered to human beings or animals to make a medical diagnosis or to restore, correct, or modify the physiological functions in human beings or animals | Pharmacy and Medicines Regulatory Authority Act, 2019 |
|  | Is there a definition for AMs/AM agents or any other variations of this term? | No |  |
|  | **Competent authority** |  |  |
|  | What is (are) the competent authorities for: (i). The authorization and management of VMPs; (ii). VMPs monitoring and enforcement; (iii). Ensuring VMPs quality, safety, and efficacy. | The Regulatory Authority shall grant pharmaceutical licenses and marketing authorizations, inspect pharmaceutical premises, regulate, and control the manufacture, importation, exportation, distribution, and sale, the advertising and promotion, of medicines and allied substances and veterinary products | Pharmacy and Medicines Regulatory Authority Act, 2019 |
|  | **Registration/authorization of veterinary AMs** |  |  |
|  | Is there a list of AMs or a registry of AMs approved by the government? | Yes | PMRA Retained Products Register |
|  | Is there legislation that prohibits the production, importation, distribution, supply, and use of VMPs unless they are authorized/registered according to national legislation?  Are there any exceptions to the rule? Under which circumstances? | Yes | Pharmacy and Medicines Regulatory Authority Act, 2019 |
|  | Does legislation include some reference or mechanism to ensure transparency in  decision-making? | Yes | Pharmacy and Medicines Regulatory Authority Act, 2019 |
|  | Does legislation include a reference to the need to coordinate the authorization/registration of veterinary AMs with the authorization of human AMs? | Yes | Pharmacy and Medicines Regulatory Authority Act, 2019 |
|  | Is there any provision on the need to restrict the authorization/use of AM critical for human purposes for veterinary use? | Section (43). “No person shall use any veterinary medicine for the treatment of human beings” | PMPB Regulation 1998 |
|  | Which requirements are in place for the authorization of AMs? (data requirement, efficacy tests, product indications (usage) and claims, package and labelling requirements, ADI, drug withdrawal times, stability when mixed with feed or drinking water, safety requirements, including potential effects on the intestinal flora of  humans)? | Cover letter, Completed, signed, and dated FORM 8A application form, certificate of pharmaceutical product (CPP), certificate(s) of suitability of the European Pharmacopoeia (CEP), compliance with current Good Manufacturing Practices (cGMP), Product information, Regional summaries, Samples, Quality overall summary, Clinical study reports and Non-clinical study reports | Guidance on submission of documentation for  Registration of a multisource (generic) finished  Pharmaceutical product (FPP) (PMRA-GD-REG-001-00) |
|  | Are these requirements included in legislation that can be easily modified by the national authority to adapt to scientific changes? (secondary legislation) | Yes | Guidance on submission of documentation for  Registration of a multisource (generic) finished  Pharmaceutical product (FPP) |
|  | Does legislation include a requirement that VMPs are classified according to typology, potential hazard and requirements related to prescription and supply? | Section 63. The categories of medicines to which this Part applies are—  (a) prescription-only medicine.  (c) pharmacy-initiated medicine.  (d) pharmacy medicine; and  (e) general sale or over-the-counter medicine. | Pharmacy and Medicines Regulatory Authority Act, 2019  PMPB Regulation 1998 |
|  | **Essential medicines list** |  |  |
|  | Is there legislation that refers to the approval of an essential medicines list21 and/or a  list of essential medicines for veterinary purposes? | Not indicated |  |
|  | Does legislation include provisions on the restriction of certain AMs to human use only?  Does it include restrictions to use essential AMs for veterinary use only as last resort and in individual treatments? | Not indicated |  |
|  | **Quality control** |  |  |
|  | Does legislation regulate the quality, safety, and efficacy control of VMPs, including standard setting, monitoring, and control (sampling, tests) before registration? | Yes | Pharmacy and Medicines Regulatory Authority Act, 2019 |
|  | Does it mandate the competent authority to do monitoring and control of VMPs post registration and in the market (pharmacovigilance) including surveillance for adverse effects, to ensure AM agents and the VMP containing them are manufactured to the appropriate quality and purity to guarantee their safety and efficacy, as well as arrangements for recall? | Yes | Pharmacy and Medicines Regulatory Authority Act, 2019  PMRA guidelines for recall of medicines from distribution system |
|  | Does legislation include specific provisions to prevent, monitor and control substandard or falsified veterinary medicinal products?  Are there specific provisions for substandard or falsified medicinal products (for human or veterinary purposes)? Or, if this is not the case, are this part of general products legislation? | Yes (General product legislation) | Guidance on submission of documentation for  Registration of a multisource (generic) finished  Pharmaceutical product (FPP)  PMRA-GD-REG-001-00 |
|  | What is the body of inspectors in charge of enforcing legislation on substandard or falsified VMPs? If these are not veterinary inspectors, does legislation provide for the need to coordinate the fight against substandard or falsified products with veterinary inspectors? | Pharmacy and Medicines Regulatory Authority, Inspectorate department | Pharmacy and Medicines Regulatory Authority Act, 2019 |
|  | Does legislation include a provision on the designation of official laboratories (either public or private, within or outside the country) to test drugs for quality and efficacy to identify substandard or falsified drugs? | The National Medicines Quality Control Laboratory | Pharmacy and Medicines Regulatory Authority Act, 2019 |
|  | **Labelling, packaging, and advertising** |  |  |
|  | Is there legislation on the labelling of AMs that specifies their authorized use, including the species for which they are authorized and the authorized route of administration, recommended dosages, storage requirements, withdrawal periods and other elements as included under III. 1) (b) of this document (Labelling) | Yes | Guidance on submission of documentation for  Registration of a multisource (generic) finished  Pharmaceutical product (FPP), PMRA-GD-REG-001-00, PMPB Regulation 1998 |
|  | Is there legislation on the inclusion of claims (such as health claims) in the packaging? | Yes | PMPB Regulation 1998 |
|  | Is there legislation on the advertising and/or marketing of VMPs? Are false or  misleading claims prohibited? | Yes | Guidelines on advertisements/ promotions of medicines, and allied substances |
|  | Are there restrictions on advertising restricted use VMPs only to veterinary  professionals and not to the public? | A medicine or allied substance which is sold by prescription only shall not be advertised to the general public. Section 68.3 | Pharmacy and Medicines Regulatory Authority Act, 2019 |
|  | Does the legislation require inclusion of expiration dates on the label? | Yes | PMPB Regulation 1998, Guidance on submission of documentation for Registration of a multisource (generic) finished Pharmaceutical product (FPP) |
|  | Does the legislation require that labels be printed in local languages? | “Every medicinal product label shall be printed in clear and indelible letters in the English language, or any other language as may be directed or approved by the board” | PMPB Regulation 1998 |
|  | Does the legislation require that the label or package specifies that the VMP is for  animal use only? | “Every medicinal product must bear the category of distribution of the medicinal product which may be represented by words or symbols as set out in the third schedule” | PMPB Regulation 1998 |
|  | **Prescription** |  |  |
|  | Does legislation prohibit the sale or dispensing of AMs (or a selected list of AMs) without prescription? | Section 65. A person shall not sell or supply medicine which is required to be sold by prescription only to any person without a Prescription. All prescriptions shall specify the medicine to be administered by reference to the generic name of that medicine. | Pharmacy and Medicines Regulatory Authority Act, 2019 |
|  | Does legislation specify that only veterinarians (or authorized professionals) can  prescribe AMs? Is this restriction specific in that such persons may only issue  prescriptions to animals under their care on the basis of a veterinary diagnosis? | “Authorized prescriber” means a medical doctor, a dental  surgeon, a veterinary surgeon, clinical officers, and medical officers registered with the Medical Council of Malawi and trained nurses and midwives registered with the Nurses and Midwives Council of Malawi | Pharmacy and Medicines Regulatory Authority Act, 2019 |
|  | Are veterinarians (or authorized professionals) mandated to only prescribe AMs for animals under his/her direct care?23 [note that this could be included under VMPs legislation, animal health or veterinary profession/statutory body legislation] | Not indicated |  |
|  | Are veterinarians held responsible and accountable for their prescription and use of  AMs? | Section 84. A person who is in charge of managing medicines and allied substances in a public health facility and who knowingly issues a false prescription in order to have medicines dispensed or used in the dispensing of medicines, commits an offence and shall, upon conviction be liable to a fine of K1,000,000 and imprisonment for twelve months. | Pharmacy and Medicines Regulatory Authority Act, 2019 |
|  | Are veterinarians required to keep records and report information on the prescription of AMs? Does the competent authority (or the veterinary statutory body) have the mandate and capacity to request this information from all veterinarians (public and private)? Are there any provision relating to confidentiality of such records and how such data can be used by the authorities or other persons with access? | The supplier of the prescription shall, on the day of which a prescription is dispensed the record in the manner accepted to the board, a complete copy of the prescription. Section 32 | PMPB Regulation 1998 |
|  | Are there any provisions that allow the off-label or extra-label use of veterinary medicinal products? If so, under what conditions? | Not indicated |  |
|  | **Sale** |  |  |
|  | Where can AMs be sold? Can AMs be sold only in pharmacies and veterinary clinics or also, in other establishments (such as feed distributors, supermarkets, or pet shops)? Is there is any differentiation in the types of AMs that can be sold by the different types of sellers? | Section 67. A person shall not sell by retail or otherwise supply medicine in a place other than a registered pharmacy practice premises, health facility or an animal health facility, except with the written approval of the Authority. | Pharmacy and Medicines Regulatory Authority Act, 2019 |
|  | Is a specific license/permit or other authorization process required for a person to sell AMs? | Section 62.A person who intends to place on the market, advertise, market, manufacture, sell, import, supply, administer or deal in any manner with any medicine or allied substance shall apply to the Authority for a marketing authorization in the prescribed manner. | Pharmacy and Medicines Regulatory Authority Act, 2019 |
|  | Can AMs be sold directly by veterinarians? Is this regulated? If so, are there mechanisms to safeguard against potential conflict of interest? | Section 67. A person shall not sell by retail or otherwise supply medicine in a place other than a registered pharmacy practice premises, health facility or an animal health facility, except with the written approval of the Authority. | Pharmacy and Medicines Regulatory Authority Act, 2019 |
|  | Are pharmacies and other establishments or professionals that sell AMs legally required to keep and report records of the sale of AMs? Does this requirement explicitly state what information must be recorded? | Every person who dispenses any medicinal product shall keep a record of such dispensing for a period of 2 years and shall preserve such record on the premises in which the dispensing takes place. Section 33 | PMPB Regulation 1998 |
|  | To whom can AMs be sold? Are there any restrictions? | Section 65. A person shall not sell or supply medicine which is required to be sold by prescription only to any person without a Prescription. All prescriptions shall specify the medicine to be administered by reference to the generic name of that medicine. | Pharmacy and Medicines Regulatory Authority Act, 2019 |
|  | Is there a prohibition to sell any of the following: unlabeled, unregistered, substandard or falsified products? | Section 98: A person shall not, during any business carried on by him, manufacture, import, assemble, dispense, sale any medicinal product or medical device which is falsified or substandard | Pharmacy and Medicines Regulatory Authority Act, 2019 |
|  | Are there provisions or reference to standards on the storage of AMs? | Yes, at all levels (manufacturer, wholesale, and retail) | PMPB Regulation 1998 |
|  | Are there requirements on safe disposal for unused and expired VMPs? | Section 27. Return of expired Ams to the manufacturer | PMPB Regulation 1998 |
|  | Does legislation contain penalties associated with selling VMPS in a manner contrary to the law? Are there specific provisions making it illegal to sell VMPs without the required prescription, or to sell counterfeit or substandard VMPs? | Part 6: Offenses and penalties | Pharmacy and Medicines Regulatory Authority Act, 2019 |
|  | **Use** |  |  |
|  | Are there rules on how AMs can be used? | Section 43: No person shall use any veterinary medicine for the treatment of human beings | PMPB Regulation 1998 |
|  | Are there any provisions on oversight or supervision by a veterinarian or other veterinary professional in the administration or use of certain VMPs? | Not indicated |  |
|  | Are therapeutic and non-therapeutic uses of AMs differentiated? How are they defined? | Not indicated |  |
|  | Is there a prohibition or restriction to use AMs for non-therapeutic purposes such as for growth promotion or productivity? Does legislation point to any protocols for risk criteria for diagnosis for disease prevention uses in animals and agriculture? | Not indicated |  |
|  | Is there any restriction in legislation [or codes of conduct?] that restrict the use of medically important AM (CIAs) in animals? | Section 43: No person shall use any veterinary medicine for the treatment of human beings | PMPB Regulation 1998 |
|  | Are the terms prevention, control and treatment defined in national legislation? | **“treatment”** in relation to disease, includes anything done or provided for alleviating the effects of the disease, whether it is done or provided by way of a cure or not | Pharmacy and Medicines Regulatory Authority Act, 2019 |
|  | Are there any limitations on the use of AMs for any therapeutic purposes? Are they limited based on a risk assessment? | Section 43: No person shall use any veterinary medicine for the treatment of huma beings | PMPB Regulation 1998 |
|  | Does legislation require AMs be administered to animals by a veterinarian or under the supervision of a veterinarian or by other authorized persons? | Not indicated |  |
|  | Are livestock/aquaculture producers legally required to keep records of the AMs they use and to report them to the national authorities? And to permit and facilitate the taking of samples? | Not indicated |  |
|  | Are livestock/aquaculture producers legally required to return unused or obsolete AMs? | Not indicated |  |
|  | Are there other legal provisions related to the disposal of unused or obsolete AMs? | Not indicated |  |
|  | **Manufacturing** |  |  |
|  | Is there legislation on production procedures and requirements for the pharmaceutical industry to ensure quality standards? [this could be included in licenses to manufacturers/pharmaceutical companies] | Yes | Pharmacy and Medicines Regulatory Authority Act, 2019 |
|  | Is there a system of registration or authorization for VMPs manufacturers  (pharmaceutical companies)? | Yes | Pharmacy and Medicines Regulatory Authority Act, 2019 |
|  | Are operators manufacturing VMPs legally required to keep records of the AMs they produce and to report them to the national authorities? | Yes | Pharmacy and Medicines Regulatory Authority Act, 2019 |
|  | Is there legislation on production procedures and requirements for the pharmaceutical industry to minimize environmental contamination with AMs resulting from the production process? | Yes | PMRA Guidelines for destruction of medicines and allied substance |
|  | Is there legislation prohibiting or restricting repackaging of AMs? | Section 67: Any medicine sold in a place other than those prescribed under subsection, shall be sold in the original package labelled by the  manufacturer | Pharmacy and Medicines Regulatory Authority Act, 2019 |
|  | Is there legislation on how to treat waste resulting from the production of AMs? | Yes | Environment Management (Waste Management and Sanitation) Regulations, 2008 |
| 2 | **Animal health and production practices to prevent animal disease in terrestrial and**  **aquatic animals** |  |  |
|  | **Animal Health** |  |  |
|  | Is there legislation that identifies the competent veterinary authority and gives it the mandate to implement the legislation?  Does this legislation recognize or facilitate the implementation of the “chain of command”? | Yes | Veterinary and Para-veterinary Practitioners Act, 2001  National Livestock Development Policy (2021-2026) |
|  | Does legislation contain a clause on the possibility for the national veterinary authority to delegate some functions? | No |  |
|  | Does the veterinary authority have the mandate to restrict or regulate the movement of animals and animal products? (Including movement permits, declaration of areas as infected areas, control areas and free areas (compartmentalization), etc.) | Yes | Control and Diseases of Animals Act, 1967  Local government (Control of animals) by-laws, 2018  Control and Diseases of Animals Regulations |
|  | Does the veterinary authority have the mandate to approve surveillance plans, including sampling and analysis?  Is there an obligation for laboratories to share data on surveillance with other reference, official and authorized laboratories, or government entities? | Yes | National Livestock Development Policy (2021-2026) |
|  | Does the veterinary authority have the mandate to approve other animal health measures, including control plans, quarantine, compulsory treatment or vaccination, animal culling and compensation?  Does legislation specify that these measures must be risk based? | Yes | National Livestock Development Policy (2021-2026) |
|  | Does legislation include the mandate of the national veterinary authority to approve a list of notifiable diseases based on the OIE list of diseases?25  Does legislation contain the obligation of notification of notifiable diseases? | Yes | National Livestock Development Policy (2021-2026) |
|  | Does the veterinary authority have the mandate to establish an early warning system, prepare a contingency plan and undertake emergency action for diseases and pathogen outbreaks? | Yes | National Livestock Development Policy (2021-2026) |
|  | Does the veterinary authority have the mandate and the possibility to declare an animal health emergency and to adopt and implement risk-based emergency measures? | Yes | National Livestock Development Policy (2021-2026) |
|  | Does legislation recognize the responsibility of farmers to maintain the health status of their animals, keep records, notify potential diseases, and implement biosecurity measures? | Yes | Guide to agriculture production and natural resources management in Malawi (2021) |
|  | Does legislation establish a system for animal identification and traceability? | Yes | National Livestock Development Policy (2021-2026) |
|  | Does the veterinary authority have the mandate to monitor and enforce veterinary legislation, including the powers of veterinary inspectors to enter private properties, take samples and review records? | Yes | National Livestock Development Policy (2021-2026) |
|  | Does the veterinary authority have the mandate to designate and regulate reference, official and authorized laboratories? | Yes | National Livestock Development Policy (2021-2026) |
|  | Does legislation contain provisions on the sharing of information and data among laboratories and between these and the surveillance authorities? Does the competent authority have the right to request for AMR-related information to reference, official and authorize laboratories, as well as other entities undertaking AMR surveillance? | Yes | Antimicrobial Resistance Strategy (2017-2022) |
|  | Does legislation give the national veterinary authority the mandate to approve import requirements that are risk based, and/or take into consideration the international reference standards? Does it require that the international trade of animals and animal products be accompanied by an international veterinary certificate issued by the national veterinary authority? | Yes | National Livestock Development Policy (2021-2026) |
|  | Is there national legislation on veterinary professions that specifies who can practice veterinary medicine, and the obligations and responsibilities of veterinarians?  Does this legislation refer to the qualification necessary to prescribe AMs? | Yes | Veterinary and Para-veterinary Practitioners Act, 2001  Pharmacy and Medicines Regulatory Authority Act, 2019 |
|  | **Animal production** |  |  |
|  | Is there animal welfare legislation, including good husbandry practices? | Animal Welfare Guidelines (Livestock, Working Animals and Companion Animals), Version 1.0 (2019) | Protection of Animals Act, 1944, Animal Welfare Guidelines (Livestock, Working Animals and Companion Animals), Version 1.0 (2019), Lilongwe City Council by laws, Control of animals, 2018 |
|  | Are there legal requirements for farmers on unused AMs? | Not indicated |  |
|  | Are there Good Husbandry Practices (GHP), Good Production Practices (GPP) or guidelines for farms, slaughterhouses, wet markets, healthcare facilities and veterinary care facilities to minimize the transmission of microbes or contamination with AMs?26 Are these reflected in legislation? | Farms  Slaughterhouses  Wet markets  Healthcare facilities and veterinary care facilities | Control and Diseases of Animals Act, 1967, Slaughter of Cattle Act, 1969, Meat Inspection Regulations, 2003, Public Health Act, 1948, Veterinary and Para-Veterinary Practitioners Act, 2001, Lilongwe City Council by laws, Control of animals, 2018, Minimum Requirements to Qualify for Veterinary Hospital/Clinic, Operating License, 2023 |
|  | Are there GPP/GHP for fish/meat/dairy processing units to minimize the transmission of microbes or contamination with AMs? | Fish  Meat  Dairy | Fisheries Conservation and Management Act, 1997  Meat and Meat Products Act, 1975  Meat Inspection Regulations, 2003  Milk and Milk Products Act,1971 |
|  | More specifically, are there GPP/GHP that recommend or require: |  |  |
|  | (i) Prohibition on the use of manure from animals being treated with AMs as fertilizer | Not indicated |  |
|  | (ii) Prohibition on the use of products or by-products from animals being treated with AMs for animal production (such as milk for young animals); | Not indicated |  |
|  | (iii) Good practice on the disposal of animal products and by-products from animals treated with AM, as well as water and other waste resulting from cleaning treated animal production premises | Yes | Public Health Act, 1948 |
|  | (iv) Appropriate storage and disposal of AMs in the farm | Not indicated |  |
|  | (v) Good practices on how to use AMs in drinking water systems | Not indicated |  |
|  | Is there legislation on health and safety at the workplace that would be applicable to farms? Could this legislation serve to protect farmers from the risks associated to exposure to Ams and AMR bacteria? | Not indicated |  |
| 3 | **Feed legislation** |  |  |
|  | **Medicated feed** |  |  |
|  | Under which legislation is medicated feed regulated? | Fertilizers, Farm Feeds and Remedies Act | Fertilizers, Farm Feeds and Remedies Act, 1970 |
|  | Is there a definition of medicated feed? Does this include, (or expressly exclude) all or some  antiparasitics? | “Farm feed” means (b) any stock lick or substance which can be used and is used as a stock lick, whether or not such stock lick or substance possesses medicinal properties | Fertilizers, Farm Feeds and Remedies Act, 1970 |
|  | Is medicated feed subject to similar requirements (of authorization, prescription, sale and use) than regular medicinal products? | Not indicated |  |
|  | Are there prohibitions or restrictions to the production, sale, import and use of medicated feed? | Not indicated |  |
|  | Is the use of medicated feed for non-therapeutic purposes (such as growth promotion) prohibited or restricted? | Not indicated |  |
|  | Is there legislation that prohibits the dispensing or sale of medicated feed in feed mills or feed establishments without a prescription from a veterinarian? | Not indicated |  |
|  | Are there specific requirements (in connection with the licensing and registration process or  other aspect) relating to manufacture or production relating to the mixing of AMs/ VMPs into feed? | Where a farm feed contains urea, biuret, antibiotics or drugs, the label shall bear—  (a) a statement giving instructions for use; and  (b) an appropriate warning. | Fertilizer, farm feeds and remedies regulations, 1996 |
|  | Are there provisions to prevent contamination from medicated feed (or feed containing AM substances) to other feed products? (e.g. cleaning methods, double production lines etc) | Not indicated |  |
|  | Does medicated feed require prescription by a veterinarian? Does medicated feed require oversight or administration by a veterinary professional? | Not indicated |  |
|  | Are there record-keeping obligations related to the prescription and use of medicated feed? | Not indicated |  |
|  | What are the labelling requirements for medicated feed? Are there provisions that require specific instructions and information be given for the correct and safe use of medicated feed? | Where a farm feed contains urea, biuret, antibiotics or drugs, the label shall bear—  (a) a statement giving instructions for use; and  (b) an appropriate warning. | Fertilizer, farm feeds and remedies regulations, 1996 |
|  | **Feed additives** |  |  |
|  | Is there a definition of feed additive or just “additive”? | “additives” means any substance added to a compound or a protein concentrate in the course of manufacturing for some specific purpose other than as a direct source of nutrient | Fertilizer, farm feeds and remedies regulations, 1996 |
|  | Is there a regulatory mechanism to approve, authorize, restrict, or prohibit the use of additives in feed production? | Fertilizer, farm feeds and remedies regulations, 1996 | Fertilizer, farm feeds and remedies regulations, 1996 |
|  | Are claims of “health” or “growth” allowed in legislation for feed additives that are not VMPs but that may have a positive impact on animal health (such as probiotics)? If so, what are the requirements to allow for these claims? | Not indicated |  |
|  |  |  |  |
| 4 | **Pesticides** |  |  |
|  | Does the country have legislation on the authorization and registration of pesticides? What is the scope of such legislation? | Yes | Pesticides Act and Regulations chapter 35:03 |
|  | Is the scope of the law broad enough to cover pesticides used for public health and  livestock production purposes (insecticides and antiparasitics)? | Yes | Pesticides Act and Regulations chapter 35:03 |
|  | For the registration or authorization of pesticides, do the authorities have the possibility to take AMR considerations into account in their decision making? *[There might not be something specific, but if decision making can take into consideration different criteria, that would be sufficient]*. Are there provisions on pre-harvest intervals following pesticide application? | Yes | Pesticides Act and Regulations chapter 35:03 |
|  | Are there licensing mechanisms related to specific pesticides activities (such as the sale, import, export, transport or special uses of pesticide (such as drift control in aerial fumigation))? | Yes | Pesticides Act and Regulations chapter 35:03 |
|  | Are there provisions on pesticide labelling? Do they follow the recommendations of the  Globally Harmonized System of Classification and Labelling of Chemicals (GHS)31? | Yes | Pesticides Act and Regulations chapter 35:03 |
|  | Are there requirements for the advertisement of pesticides? | Yes | Pesticides Act and Regulations chapter 35:03 |
|  | Are there requirements for the management and disposal of used pesticide containers? | Yes | Pesticides Act and Regulations chapter 35:03 |
|  | Are there requirements for the safe disposal of unused or obsolete pesticides? | Yes | Pesticides Act and Regulations chapter 35:03 |
|  | **5) Food safety** |  |  |
|  | **Food safety system** |  |  |
|  | Is there national legislation governing food safety? Which legal instruments regulate food  safety? | Yes | Public Health Act 1948  Malawi Bureau of Standards Act 1972:2012  Fisheries Conservation and Management Act 1997  Meat and Meat Products Act 1976  Milk and Milk Products Act 1971  PMRA, 2019  Iodization of Salt Act 1995  Consumer Protection Act 2003  Veterinary and Para-veterinary act, 2001 |
|  | Does legislation establish a competent national authority or authorities to coordinate food safety in all stages of the food production chain? | 1. Malawi Bureau of Standards 2. Pharmacy, Medicine, and Regulatory Authority 3. Board of Veterinary surgeons 4. Consumer protection council | 1. Malawi Bureau of Standards Act 1972:2012 2. PMRA, 2019 3. Veterinary and Para-veterinary act, 2001 4. Consumer Protection Act 2003 |
|  | Does legislation contain a provision on the possibility for this authority to delegate some functions? | No |  |
|  | Which is (are) the national institution(s) with a mandate on food safety across the different  stages of the food chain? | Malawi Bureau of Standards  Pharmacy, Medicine, and Regulatory Authority  Board of Veterinary surgeons  Consumer protection council | Malawi Bureau of Standards Act 1972:2012  PMRA, 2019  Veterinary and Para-veterinary act, 2001  Consumer Protection Act 2003 |
|  | Is there a mechanism for coordination across the different institutions with a role on food safety? | No |  |
|  | Does the national authority (or the authorities) have the mandate to approve measures to  prevent, identify, and control food hazards? (that may include microbial or AM contamination) | Yes | Malawi Bureau of Standards Act 1972:2012 |
|  | Are they mandated to approve, monitor, and control food safety and quality standards based on Codex standards? | Yes | Malawi Bureau of Standards Act 1972:2012  Certification Marks Regulations of MBS |
|  | Does legislation recognize the primary responsibility of food operators for food safety? Are there mechanisms to keep food operators under regulatory control (such as registration, licenses or permits)? | Yes | Malawi Bureau of Standards Act 1972:2012  Certification Marks Regulations of MBS |
|  | Does the food safety authority have the mandate to conduct surveillance programs to identify food hazards? (including microbiological contamination) | Yes | Malawi Bureau of Standards Act 1972:2012 |
|  | Does the food safety authority have the legal mandate to designate and regulate reference, official and authorized laboratories? | Yes | Malawi Bureau of Standards Act 1972:2012 |
|  | Is there an obligation for laboratories to share surveillance data with other reference, official and authorized laboratories, or other entities undertaking AMR surveillance? | Yes | Antimicrobial Resistance Strategy (2017-2022) |
|  | Does the competent authority have the right to request for AMR-related information to reference, official and authorize laboratories, as well as other entities undertaking AMR surveillance? | Yes | Antimicrobial Resistance Strategy (2017-2022) |
|  | Does legislation include provisions of food traceability and recall? Does it include references to a rapid alert system to declare a food outbreak/emergency and to adopt risk-based food safety measures to contain the outbreak? | Yes | Malawi Bureau of Standards Act 1972:2012 |
|  | Does national legislation provide the mandate to the food safety authority(ies) to approve risk-based import requirements? And to certify food for export? | Yes | Malawi Bureau of Standards Act 1972:2012 |
|  | **MRLs** |  |  |
|  | Is there legislation on the monitoring and control of MRLs of VMPs and pesticides in food? | Yes | Malawi Bureau of Standards Act 1972:2012 |
|  | Is it compulsory to set up MRLs for all authorized AMs? | Yes | Malawi Bureau of Standards Act 1972:2012 |
|  | Are MRLs aligned with Codex? | Yes | Malawi Bureau of Standards Act 1972:2012 |
|  | **Food additives** |  |  |
|  | Is there a regulated procedure to approve food standards (please describe)? Does this procedure indicate that food standards should be based on Codex standards? | Yes | Malawi Bureau of Standards Act 1972:2012  Certification Marks Regulations of MBS |
|  | Does the competent authority have the capacity to prohibit, restrict or control the use of a food additive at any time? | Yes | Malawi Bureau of Standards Act 1972:2012 |
|  | **Environment, Soil and Waste** |  |  |
|  | In environmental protection or waste legislation, are there mechanisms that enable the authority to regulate wastewater and/or waste disposal? | Yes | Environment Management (Waste Management and Sanitation) Regulations, 2008 |
|  | Is there specific legislation or standards regulating the disposal of waste (including wastewater) from hospitals, medical centers, or other establishments where AMs are produced, stored or used? | Yes | Environment Management (Waste Management and Sanitation) Regulations, 2008 |
|  | Is there specific legislation or standards regulating the disposal of waste from farms, animal markets, slaughterhouses, quarantine facilities and other establishments where animals might be produced or kept? | Yes | Environment Management (Waste Management and Sanitation) Regulations, 2008 |
|  | Is there legislation where the authorities can approve certain requirements or criteria for registration of farms (or approval to carry out other activities) that are environment-related?  Do they have to present an environmental impact assessment (EIA) for registration? | Yes | Environment Management (Waste Management and Sanitation) Regulations, 2008 |
|  | Is there legislation on soil quality, monitoring residues in soil or prevention of soil and water contamination that would cover contamination with AMs? | Yes | Environment Management (Waste Management and Sanitation) Regulations, 2008 |
|  | Are there regulations on antibiotic residues in effluents and waste from industries, mills, farms and other animal gathering businesses? | Yes | Environment Management (Waste Management and Sanitation) Regulations, 2008 |
|  | **Water quality** |  |  |
|  | Is there legislation controlling water quality, drinking water quality, environmental water quality and/or recreational water quality? | Yes | Waterworks Act, 1996  Water Resources Act, 2013  Water Resources Regulations, 2018  Fisheries Conservation and management regulations, 2000 |
|  | Is there legislation to control the quality of water used in agriculture, including aquaculture? | Yes | Water Resources Regulations, 2018 |
|  | Are there legislation or standards concerning the reuse of wastewater for agricultural purposes? | Not indicated |  |
|  | Is there legislation on the use of AMs in aquaculture establishments in open water or in flowthrough systems? | Not indicated |  |
|  | Is there legislation that places restrictions on the types of activities (industrial, agricultural) that can be carried out in or near water sources? | Yes | Water Resources Regulations, 2018 |
|  | Is there legislation controlling the types of discharges and pollutants that can be released into freshwater resources, including watercourses, lakes and aquifers? Could this be used to introduce AMR considerations? This may be already covered in section 6 on the environment and waste, but if such provisions have not been identified in section 6, please check for relevant provisions in water and water quality-related legislation | Yes | Waterworks Act, 1996  Water Resources Act, 2013  Water Resources Regulations, 2018  Fisheries Conservation and management regulations, 2000 |
|  | **Plant Health** |  |  |
|  | Does the country have national legislation implementing the International Plant Protection Convention (IPPC) and its International Standards for Phytosanitary Measures (ISPMs) ? | Yes | Plant protection act, 2018  Plant protection regulations (Import, Export, Fumigation)  Noxious weed regulations, 1951  Seed regulations, 2018  Pesticide regulations, 2002 |
|  | More specifically, is there a designated National Plant Protection Organization (NPPO)36 or responsible body for plant protection? | Plant Protection Unit, the Department  of Agricultural Research Services | Plant protection act, 2018 |
|  | Is the NPPO or responsible body for plant protection mandated to regulate and control plant pests both internally (in cultivated plants and wild flora) and in the international consignments of plants, plant products or other regulated articles 37 through surveillance, pest risk analysis, import, export and transit control, treatments, inspections, designation of areas as free from a pest, infected areas or areas of low pest prevalence? | Yes | Plant protection act, 2018 |
|  | Is the NPPO or responsible body for plant protection mandated to issue phytosanitary certificates and ensure the phytosanitary security of the consignment upon delivery? | Yes | Plant protection act, 2018 |
|  | Does plant health legislation require that plants, plant products and regulated articles imported into the country undergo a pest risk analysis prior to import? Does it require that technically justified phytosanitary requirements are put in place and, if necessary, that consignments are  accompanied by a valid phytosanitary certificate issued by the NPPO or responsible body for plant protection of the exporting country? | Yes | Plant protection act, 2018  Plant protection regulations (Import, Export, Fumigation) |
|  | Is the NPPO or responsible body for plant protection mandated to elaborate a list of regulated pests and to approve control programs to control such pests? | Yes | Plant protection act, 2018 |
|  | Is the NPPO or responsible body for plant protection mandated to have contingency plans in place? Is it mandated to designate reference, official and authorized laboratories? | Yes | Plant protection act, 2018 |
|  | Is the NPPO mandated to designate reference, official and authorized laboratories? Is there an obligation for laboratories to share surveillance data with other reference, official and authorized laboratories, or government entities? | Yes | Plant protection act, 2018 |
|  | Does legislation contain a clause on the possibility for the NPPO or responsible body for plant protection to delegate some functions? Are there other agencies that have been mandated to perform the NPPO functions and, if yes, are those functions still under the primary responsibility of the NPPO? | No |  |
|  | Does legislation contain some reference to the need for coordination with other institutions (such as customs)? | Yes | Plant protection act, 2018 |
|  | **Institutional coordination** |  |  |
|  | Is there any regulatory instrument setting up a coordination mechanism across ministries and other entities for AMR governance? (horizontal level) | No, just a strategy | Antimicrobial Resistance Strategy (2017-2022)  National Livestock Development Policy (2021-2026) |
|  | If so, does it include all areas relevant for AMR? (consider the areas included in this report as reference)? | The strategy alone is present | Antimicrobial Resistance Strategy (2017-2022) |
|  | How are the composition, mandate and decision-making powers of the AMR coordination mechanism defined? | The key oversight functions of the AMR activities is managed by the Minister of Health (MoH) through the AMR National Coordinating Centre, whose duties are defined by AMR Technical Working Group. As a government agency, the MoH, through the AMR National Coordinating Centre, will set the agenda for AMR surveillance in Malawi in collaboration with key stakeholders. The MoH has an organizational structure, with establishment for both technical and administrative staff. Each of the established positions has specified job descriptions. Several partner institutions and civil society will complement the work of the Ministry in discharging its core functions on AMR through advocacy, surveillance, research and training as well as quality assurance | Antimicrobial Resistance Strategy (2017-2022) |
|  | Does this mechanism include also representatives from the decentralized level and the private sector? | Yes | Antimicrobial Resistance Strategy (2017-2022) |
|  | If it includes private sector representatives, is there any safeguard against potential conflicts of interest? | Yes | Antimicrobial Resistance Strategy (2017-2022) |
|  | Is the country a federal/decentralized state? How does this impact on AMR management? | The systems and structures established by the Government of Malawi and those created under the partnership alignment, the coordination and the decentralization system play their defined role and responsibilities during the implementation of the AMR strategy. | Antimicrobial Resistance Strategy (2017-2022) |
|  | How are national competences shared between the central and the decentralized level for those areas relevant for AMR (health, agriculture, environment…)? | The partnership structure guides external coordination of service delivery by all stakeholders at the respective levels of care. All partners providing services at a given level of care engage with each other through this structure. | Antimicrobial Resistance Strategy (2017-2022) |
|  | Does the central level have a mechanism to approve legislation (for example based on common interest) that prevails over the legislation of the decentralized level in the areas relevant for AM and AMR? | No |  |
|  | Is there a national body established for prevention and control of AMR? | Yes, Antimicrobial Resistance Coordinating Center (AMRNCC) | Antimicrobial Resistance Strategy (2017-2022) |
|  | Are the antimicrobial resistance coordination committee’s members, functions, and decision-making powers defined in the instrument? | Yes | Antimicrobial Resistance Strategy (2017-2022) |
|  | Is the information on funding AMRNCC provided? | No |  |
|  | Is there a system that approves national legislation to be superior to the sub-national (decentralized) legislation about antimicrobials and antimicrobial resistance? | No |  |
|  | Does Malawi have a prioritized research agenda within AMR? | No |  |
|  | Is there a one health strategy against AMR from different relevant ministries or stakeholders? | Yes | Antimicrobial Resistance Strategy (2017-2022) |
|  |  |  |  |
|  | **The role of herd veterinarian** |  |  |
|  | Are veterinarians allowed to sell VMPs to make a profit? | Not indicated |  |
|  | Are all types of farms obligated to have veterinary advisory service contracts between the veterinary practitioners and the farmers? | No |  |
|  | Are there treatment guidelines for specific hosts and/or diseases? | No |  |
|  | How often are the treatment guidelines revised? | No |  |
|  | Is AMR surveillance data taken into consideration when revising these guidelines? | No |  |
|  | Are there generally unannounced farm visits for inspection? | No |  |
|  | Do veterinary practitioners revisit the livestock herds for complete registration when the gaps in farming are noticed? | Not indicated |  |
|  | Are there legal actions against the farmer such as issuance of fines in case he or she fails to comply with the rules within the stipulated time frame? | Yes | Control and Diseases of Animals Act |
|  | Is there compulsory antimicrobial susceptibility testing before prescribing any AMs? | No |  |
|  | Is there mandatory showering, changing of clothes, handwashing, and disinfection for all persons involved in the chain of animal feed production, processing, and transportation to the end consumer? | Yes | Control and Diseases of Animals Act  Swine Fever rules |
|  | Is it required that anyone who has interacted with pig herds must wash his or her hands and change clothing before leaving the herd? | Yes | Control and Diseases of Animals Act  Swine Fever rules |
|  | Are persons working in the pig industry required to study a course in hygienic practices? | Not indicated |  |
|  | Are the workers who deal with herds of pigs required to bathe at the end of the working day? Are individuals who often take short periods in pig stables required to put on disposable overalls? | Yes | Control and Diseases of Animals Act  Swine Fever rules |
|  | **Monitoring program** |  |  |
|  | Is there an integrated AMU monitoring program? | No |  |
|  | Is AMU data centrally stored in one database that is accessible by all relevant institutions? | Yes, WHONET |  |
|  | Is there AMR data centrally stored in one database that is accessible by all relevant institutions? | No |  |
|  | How is the monitoring program financed? | Not indicated |  |
|  | Is there a national target for minimizing the use of antimicrobials in animals? | No |  |
|  | How often is this target evaluated/revised? | No |  |
|  | Is there benchmarking of the veterinarian’s prescription of antibiotics? | No |  |
|  | Are farmers required to attend courses on farming best practices, biosecurity, and prudent and reduced use of AMs including updates on changes to the legislation? | No |  |

**Supplementary Material 3: Social Network Analysis of the Antimicrobial Use Governing Institutions in Malawi**

| **Label** | **Type** | **One Health Scope** | **betweenness** | **closeness** | **degree** | **reach-efficiency** | **eigenvector** | **indegree** | **metrics::last** | **micmac exposure** | **micmac influence** | **outdegree** | **reach** | **size** |
| --- | --- | --- | --- | --- | --- | --- | --- | --- | --- | --- | --- | --- | --- | --- |
| Blantyre Society for the Protection and Care of Animals | NGO | Animal | 0.0 | 0.4 | 2.0 | 0.2 | 0.0 | 1.0 | 2.0 | 0.8 | 0.6 | 2.0 | 0.5 | 3.0 |
| Basic Services Development Agency | NGO | Environment | 0.0 | 0.4 | 1.0 | 0.3 | 0.0 | 1.0 | 1.0 | 0.8 | 0.6 | 1.0 | 0.5 | 2.0 |
| Malawi Bureau of Standards | Government Department | All | 0.0 | 0.4 | 2.0 | 0.1 | 0.0 | 1.0 | 2.0 | 0.5 | 0.7 | 2.0 | 0.3 | 3.0 |
| Ministry of Education | Government Department | All | 0.0 | 0.5 | 6.0 | 0.1 | 0.1 | 5.0 | 6.0 | 0.6 | 1.0 | 4.0 | 0.9 | 7.0 |
| Kamuzu University of Health Sciences (KUHES) | Government Department | Human | 0.1 | 0.5 | 5.0 | 0.1 | 0.0 | 4.0 | 5.0 | 0.6 | 0.9 | 4.0 | 0.5 | 6.0 |
| All Creatures Animal Welfare Trust | NGO | Animal | 0.0 | 0.4 | 1.0 | 0.3 | 0.0 | 1.0 | 1.0 | 0.8 | 0.6 | 1.0 | 0.5 | 2.0 |
| Livestock Farmers Cooperatives | NGO | Animal | 0.0 | 0.0 | 4.0 | 0.0 | 0.0 | 4.0 | 4.0 | 1.0 | 0.0 | 0.0 | 0.0 | 5.0 |
| United Purpose | NGO | Animal | 0.0 | 0.4 | 1.0 | 0.3 | 0.0 | 0.0 | 1.0 | 0.0 | 0.6 | 1.0 | 0.6 | 2.0 |
| Ministry of Health | Government Department | Human | 0.3 | 0.7 | 13.0 | 0.1 | 0.1 | 8.0 | 13.0 | 0.9 | 0.8 | 11.0 | 0.9 | 13.0 |
| Clinton Health Access Initiative(CHAI) | NGO | Human | 0.0 | 0.4 | 1.0 | 0.2 | 0.0 | 1.0 | 1.0 | 0.9 | 0.7 | 1.0 | 0.5 | 2.0 |
| Ministry of Agriculture (Department of Animal Health and Livestock Development) | Government Department | Animal | 0.4 | 0.7 | 15.0 | 0.0 | 0.1 | 14.0 | 15.0 | 0.5 | 0.7 | 12.0 | 0.7 | 16.0 |
| African Institute for Development Policy (AFIDEP) | NGO | Human | 0.0 | 0.5 | 2.0 | 0.2 | 0.0 | 2.0 | 2.0 | 0.7 | 0.7 | 2.0 | 0.6 | 3.0 |
| Ministry of Natural Resources | Government Department | Environment | 0.0 | 0.4 | 8.0 | 0.1 | 0.1 | 8.0 | 8.0 | 0.9 | 0.9 | 1.0 | 0.5 | 7.0 |
| Ministry of Finance | Government Department | All | 0.0 | 0.5 | 7.0 | 0.1 | 0.1 | 6.0 | 7.0 | 0.8 | 0.5 | 4.0 | 0.7 | 8.0 |
| Malawi-Liverpool Wellcome Trust | NGO | All | 0.0 | 0.5 | 4.0 | 0.1 | 0.0 | 3.0 | 4.0 | 0.5 | 1.0 | 3.0 | 0.6 | 5.0 |
| AMR National Coordinating Center (AMRNCC) | Government Department | All | 0.3 | 0.7 | 14.0 | 0.1 | 0.1 | 8.0 | 14.0 | 0.7 | 0.7 | 14.0 | 0.9 | 13.0 |
| Malawi University Of Business And Applied Science (MUBAS) | Government Department | All | 0.0 | 0.4 | 3.0 | 0.1 | 0.0 | 3.0 | 3.0 | 0.7 | 0.9 | 2.0 | 0.3 | 4.0 |
| Ministry of Gender | Government Department | All | 0.0 | 0.6 | 6.0 | 0.1 | 0.1 | 5.0 | 6.0 | 0.7 | 0.6 | 5.0 | 0.9 | 7.0 |
| Food and Agriculture Organisation (FAO) | NGO | Animal | 0.0 | 0.6 | 7.0 | 0.1 | 0.0 | 3.0 | 7.0 | 0.7 | 0.9 | 6.0 | 0.9 | 8.0 |
| Lilongwe Society for the Protection and Care of Animals (LSPCA) | NGO | Animal | 0.0 | 0.4 | 1.0 | 0.3 | 0.0 | 1.0 | 1.0 | 0.8 | 0.6 | 1.0 | 0.5 | 2.0 |
| Pharmacy, Medicines Regulatory Authority | Government Department | All | 0.0 | 0.5 | 4.0 | 0.1 | 0.0 | 4.0 | 4.0 | 0.7 | 0.7 | 2.0 | 0.6 | 5.0 |
| Central Veterinary Lab | Government Department | Animal | 0.0 | 0.6 | 5.0 | 0.1 | 0.0 | 2.0 | 5.0 | 0.7 | 0.8 | 5.0 | 0.8 | 6.0 |
| Lilongwe University of Agriculture and Natural Resources (LUANAR) | Government Department | Animal | 0.0 | 0.5 | 6.0 | 0.1 | 0.1 | 4.0 | 6.0 | 0.7 | 0.7 | 4.0 | 0.8 | 6.0 |
| Lilongwe Wildlife Trust | NGO | Animal | 0.0 | 0.5 | 2.0 | 0.2 | 0.0 | 1.0 | 2.0 | 0.8 | 0.8 | 2.0 | 0.6 | 3.0 |
